# Nano/Microscale Thermal Field Distribution: Conducting Thermal Decomposition of Pyrolytic-Type Polymer by Heated AFM Probes

**DOI:** 10.3390/nano10030483

**Published:** 2020-03-07

**Authors:** Bo Li, Yanquan Geng, Yongda Yan

**Affiliations:** 1 Key Laboratory of Micro-systems and Micro-structures Manufacturing of Ministry of Education, Harbin Institute of Technology, Harbin 150001, China; 14B308012@hit.edu.cn (B.L.); yanyongda@hit.edu.cn (Y.Y.); 2 Center for Precision Engineering, Harbin Institute of Technology, Harbin 150001, China

**Keywords:** nanoscale thermal analysis, nano/microscale temperature field distribution, nano/microscale air gap thermal transfer, AFM, scanning thermal microscopy

## Abstract

In relevant investigations and applications of the heated atomic force microscope (AFM) probes, the determination of the actual thermal distribution between the probe and the materials under processing or testing is a core issue. Herein, the polyphthalaldehyde (PPA) film material and AFM imaging of the decomposition structures (pyrolytic region of PPA) were utilized to study the temperature distribution in the nano/microscale air gap between heated tips and materials. Different sizes of pyramid decomposition structures were formed on the surface of PPA film by the heated tip, which was hovering at the initial tip–sample contact with the preset temperature from 190 to 220 °C for a heating duration ranging from 0.3 to 120 s. According to the positions of the 188 °C isothermal surface in the steady-state probe temperature fields, precise 3D boundary conditions were obtained. We also established a simplified calculation model of the 3D steady-state thermal field based on the experimental results, and calculated the temperature distribution of the air gap under any preset tip temperature, which revealed the principle of horizontal (<700 nm) and vertical (<250 nm) heat transport. Based on our calculation, we fabricated the programmable nano-microscale pyramid structures on the PPA film, which may be a potential application in scanning thermal microscopy.

## 1. Introduction

In recent years, atomic force microscopy (AFM) based scanning thermal microscopy (SThM) has attracted widespread interest due to a range of potential applications in nanomanufacturing and nanometrology [1]. In nanomanufacturing, this technique has contributed substantially to the present nanofabrication methods due to its high efficiency, high precision, and capability to fabricate diversified functional structures [2]. This technology makes it possible to fabricate nanostructures on polymers [3,4] as well as utilize nanoscale thermochemical reactions to pattern nanostructures with special electrical [5,6], wetting [7], and optical characteristics [8] on various materials. In nanometrology, the AFM thermal probe is used to measure the real temperature of the tip–substrate interface [9], the topography of the material surface [10], and the analysis of nanoscale heat transfer [11,12]. Among the above-mentioned applications of SThM, the nanoscale thermal field distribution (NTFD) is the cornerstone of precise nanostructure fabrication or nanoscale thermal analysis, and has been a longstanding challenge as a result of the presence of scale effects. However, the limitations of the determining environment, the thermal properties of the materials and the different nanostructures on the sample surface to the NTFD methodology measurement have motivated the development of various effective measurement methods [9,13,14,15,16,17,18].

Recently, Park et al. [9] used a platinum-resistance thermometer in an AFM platform to measure the substrate surface temperature and the cantilever temperature simultaneously. Sadat et al. [16] utilized the AFM and a bottom electrode to map the NTFD in metallic films. Ge et al. [18] adopted a film calibration sample with adjustable sizes to calibrate the temperatures of the SThM probe. In their subsequent study [19], they demonstrated the heat transfer from the SThM probe to the gold wire via experiment, and suggested that in addition to the thermal properties of the materials, the geometry and feature dimensions of the sample were equally important to the thermal spreading resistance of the material. All these studies have mainly focused on the NTFD at the tip–sample interface as well as the influencing factors of the interface thermal resistance or the diffusion thermal resistance when the tip and the sample are in close contact.

Pratap et al. [20] found that the density of the air gap between the probe tip and the sample posed a significant impact on the heat conduction of the tip. Lin et al. [21] used experimental methods showing that the effect of heat conduction on the air gap was still visible when the size of the air gap was more than five microns, which proved the importance of the heat transfer of the air gap. In fact, in many nanofabrication processes using thermal probes, the effect of the thermal field around the heated tip often induces the thermal decomposition on the sample surface [22,23,24,25]. The decomposition process then causes the tip-sample heat transfer to transform from an intimate-contact interface heat transfer mode to a gas-gap heat transfer mode. In addition, when nanodevices such as data storage chips and Pirani vacuum gauges are used under service conditions, the thermal conduction of the air gap has a great influence on their performance. For example, Gotsmann et al. [26] indicated that the high-density data storage chips they designed were capable of 10-year of data retention at 85 °C. However, once the temperature of the operating environment exceeds 85 °C, it may cause thermal damage to the nanoscale hollow storage points [27,28].

Heat transfer in nano/microscale air gaps consistently exist during nanostructure fabrication through thermal decomposition, and in nanomaterial applications in a thermal environment. However, there are only a few works on the nano/microscale air gap. Nonetheless, Huang et al. [29] used micro hot plate arrays to study the heat transfer effect in the microscale air gap and found that the heat transfer in the air gap was significantly affected by the size effect. SThM technology is considered as an effective method to investigate the nano/microscale temperature distribution of the air gap. The corresponding simulation results [29,30,31,32] of the model for heat transfer between the tip and the surrounding air environment can be used for reference.

By evaluating several currently available theoretical models for heat transfers in and around thermal probes [29,30], it was concluded that establishing the model should include factors such as the geometry of the heat source of the tip and the relative position of the sample surface, which resulted in there were multiple heat transfer modes between the heated tip and the sample surface. These different heat transfer modes would then produce various degrees of influence on the heat distribution of the sample surface. King et al. [31] established a continuum finite element simulation to investigate the heat diffusion from the cantilever to the surrounding air. Hoerber et al. [32] obtained the temperature distribution around the tip–sample interface by the simulation approach. Duvigneau et al. [33] developed a 1D steady-state model to calculate the temperature profiles close to the tip–polymer contact. However, the temperature distribution in the nano/microscale tip–substrate air gap was still not available due to the limitations of the above-mentioned models. 

In this study, the pyrolytic material polyphthalaldehyde (PPA) film and AFM imaging of the nano/microscale decomposition structures (pyrolytic region of PPA) were utilized to investigate the nano/microscale temperature distribution in the air gap between the heated tip and substrate. The process of our work was twofold: (i) adopting the experimental method to directly measure the feature sizes of the decomposition structures to locate the position of the isothermal surface of the PPA decomposition temperature in the thermal field, and (ii) establishing a simplified calculation model of the 3D steady-state thermal field based on the experimental results (3D boundary conditions) and calculating the temperature distribution of the probe under any preset temperature. The results provide an experimental method for the study of temperature distribution in the nano/microscale air gap thermal field, which enriches the theory of nanoscale heat transfer and provides theoretical support for the dimension design of nanostructures fabricated on pyrolytic materials by SThM technology. It is of great significance for the further development of nano/microscale manufacturing and thermal analysis.

## 2. Materials and Methods 

### 2.1. Polyphthalaldehyde (PPA) Films

The PPA material (average M_n_ is 5000–8000, poly dispersity MW/MN < 2.0, Sigma-Aldrich Company, China) were dissolved in cyclohexanone at a concentration of 8.5 wt%. The solution was spun on a square single-crystal silicon substrate, which had been ultrasonically cleaned in acetone and then in alcohol for 10 min. The spinning speed and time were 500 rpm and 30 s, respectively, to obtain film thicknesses of ~600 nm. The PPA film was subsequently baked at 120 °C for 3 min to remove the solvent.

### 2.2. Atomic Force Microscopy

The SThL and thermal analysis measurements experiments were performed with a commercial AFM operated with a Nanoscope V controller (Dimension Icon; Bruker Corporation, Germany), equipped with heatable silicon AFM probes (VITA-DM-NANOTA-200; Bruker Company, Germany) in contact mode. Temperatures of the tip were controlled with a thermal analysis accessory (VITA^TM^; Bruker Corporation, USA). The probe temperatures were calibrated [17] with the polymer melting point standards (polycaprolactone, polyethylene, and poly(ethylene terephthalate) with melting points of 55, 116, and 235 °C, respectively).

### 2.3. Thermal Expansion Measurements

The thermal expansion of the PPA film was measured by noting the vertical deflection errors of the cantilever as a function of temperature, while *T*_t_ was ramped from room temperature to 188 °C with a rate of 7 °C·s^−1^ at the preselected position. During the measurement process, the contact load feedback was disabled. A silicon tip (MPP-11120; Bruker Company, Germany) was used to capture the AFM images of formed expansion structures using the tapping mode.

### 2.4. Heat-Induced decomposition 

The array of pyramid decomposition structures (air gaps) on the PPA film was fabricated by a heatable AFM probe through the following three steps in an atmospheric air environment: (i) The heated tip with the room temperature was positioned in contact with the PPA film surface with an initial contact force ~20 nN; (ii) the tip temperature was instantaneously raised to the preset temperature (ranging from 190–220 °C) by applying a fixed voltage to the integrated heater via the cantilever while the tip was controlled by the scanner to stay at the initial location for a specified heating duration (ranging from 0.3 to 120 s); and (iii) after stopping heating, the heated tip was cooled for 10 s to ensure that its temperature was low enough to avoid inducing decomposition [31] and then moved to the next preselected position by controlling the scanner. Subsequently, steps (ii)–(iii) were repeated until the array structure was completely fabricated. Afterward, the probe was withdrawn from the sample. Tapping mode AFM topography of the formed array air gaps was captured by using the same probe as used in the imaging of formed expansion structures. 

## 3. Results and Discussion

The PPA, a polymer material with the decomposition property under fixed temperature [34], was used to prepare a film with a thickness of >600 nm. The thermal decomposition threshold temperature (*T_d_*) of the formed PPA film is rigorous at 188 °C, which means that the PPA film surface would decompose and be gasified away as soon as its temperature reaches 188 °C.

**Figure 1:**
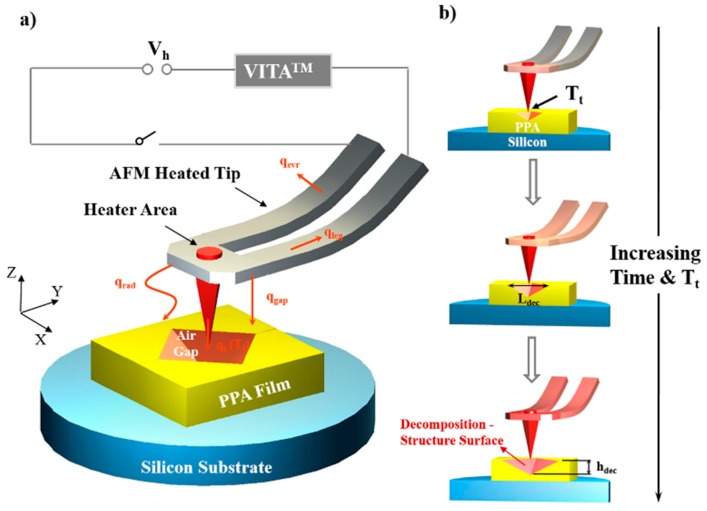
Schematic of an atomic force microscope (AFM) heated probe tip hovering above the polyphthalaldehyde (PPA) film on a silicon substrate to fabricate the pyramid decomposition structure (air gap). (**a**) The integrated heater area, lying above the tip free end, is electrified through the cantilever to heat the tip by controlling the VITA^TM^ heating module. The resulting heat fluxes are represented by the orange arrows for the different modes of heat lost: through the cantilever legs (*q_leg_*) and tip (*q*_t_) by conduction, from the tip to environment (*q*_evr_) by conduction, from the tip to PPA film (*q*_gap_) by conduction, and the radiation (*q*_rad_). The decomposition of PPA film, which causes a pyramid air gap structure to form on its surface, is induced by *q*_gap_. (**b**) The cross-section plot of the sample underneath the heated cantilever (being mounted at an angle of ~12°) showing the dimension of the air gap structure changing with the increasing tip temperature and heating time. The horizontal and vertical sizes of the air gap structure are indicated by the two double-headed arrows, which are located on and perpendicular to the PPA surface and marked by *L*_dec_ and *h*_dec_, respectively.

The decomposition structures on the PPA film surface fabricated by the heated tip were considered as nano/microscale air gaps to be used to investigate the temperature field distribution around the tip (Figure 1a). The decomposition process was only controlled by thermal transfer from the heated tip. The location of the air gap surface was exactly the location of the isothermal surface at 188 °C in the temperature field, which was the basis of our investigation. 

The pyramid air gaps underneath the heated AFM probe on the PPA film had an approximately rhombic surface contour. The decomposition-structure horizontal (*L*_dec_) as well as vertical (*h*_dec_) dimensions were considered as the feature sizes of the air gap. These were only associated with the tip temperature and heating time under a fixed relative position of the heated tip to the PPA surface. By measuring the values of *L*_dec_ and *h*_dec_, which were enlarged by increasing the tip temperature or the heating time, the location of the isothermal surface of 188 °C in the air gap temperature field could then be determined (Figure 1b).

Forms of energy dissipation of the generated heat around the integrated heater with a temperature *T*_heater_ are described in Figure 1. According to calculations conducted by Kim et al., about 20% of the total power was dissipated around the cantilever to the environment through *q*_evr_ [31], with most of the remaining total power via *q*_gap_ dissipated between the cantilever and the polymer material. In a follow-up study [9], Park et al. found that the heat accounted for about 75% of the total power. The effect of the thermal radiation on the bottom polymer material was concluded to be negligible [10,35]. The conduction of q_t_ from the tip to the material through the tip–sample interface only accounted for 0.1% of the total power; however, when there is a contact interface, due to the great interface thermal resistance, the temperature of the contact point at the tip peak is much higher than any other region of the tip [32]. Moreover, if the substrate material did not decompose upon heating, the lateral heat transport range from the contact point to the surrounding organic material could reach up to the micron level (the radius >9 μm) [33], and the remaining less than 5% of the total power dissipated via *q*_leg_. Once there exists a nanoscale air gap instead of a contact point or a contact interface, as shown in the experimental results in Figure 1, how heat affects the substrate material from the cantilever tip through the nano/microscale air gap requires a more detailed analysis.

### 3.1. Heat Induced Localized PPA Decomposition

**Figure 2:**
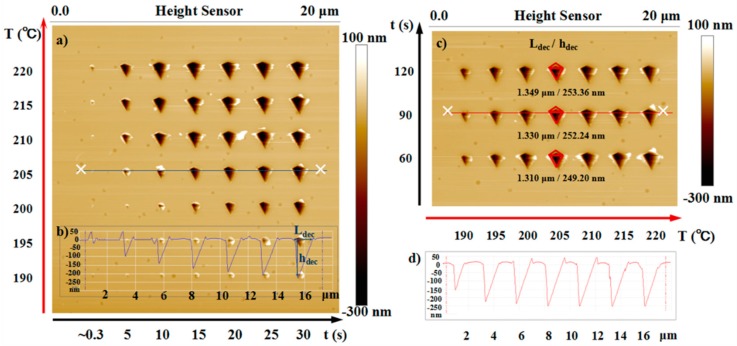
(**a**) AFM topographic image of the pyramid air gap structures on the film surface formed by using a heated tip to induce localized PPA decomposition. The corresponding tip temperatures (190–220 °C) and heating duration (0.3–30 s) for each experimental air gap are shown in the left and bottom side of the image, respectively. (**b**) The cross-section plot of the position marked in (a) by a blue line. The method for measuring the feature sizes (*L*_dec_ and *h*_dec_) of the air gap structures is shown in the right-side structure section. (**c**) AFM imaging plot of the pyramid air gap structures formed under the condition of the heating duration of 60–120 s and the tip temperature of 190–220 °C. For the tip temperature of 205 °C, the values of the two feature sizes of the structures are given, close below the corresponding structure. (**d**) The cross-section plot of the position marked in (c) by a red line.

**Figure 3:**
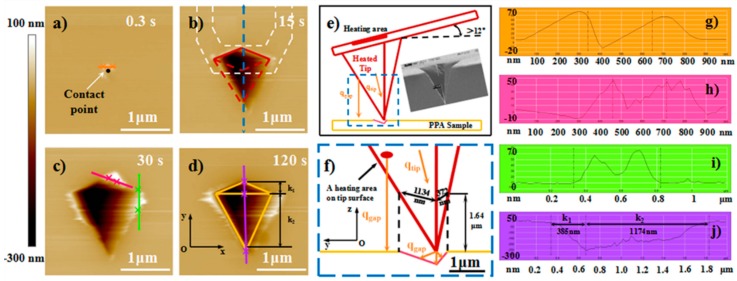
AFM topographic images of the pyramid air gap structures and the cross-section plot of the marked location. The heating times in the topographic images were 0.3 s (**a**), 15 s (**b**), 30 s (**c**), and 120 s (**d**), the tip temperature was kept at 205 °C. The height cross-section plot of the location marked by corresponding color lines are shown in (**g**), (**h**), (**i**) and (**j**), respectively. (a) The white arrow points to the initial tip-sample contact point. (b) The white dashed lines schematically show the cantilever orientation (not to scale). The area enclosed by red dashes is the deviation area of the surface contour away from a symmetric rhombus. (**e**) The cross-section plot along the blue dashed line in (b), showing the accurately geometric shape of the heated tip and the relative position of the cantilever as well as the tip and the air gap structure on the PPA surface. Scanning electron microscope (SEM) image provided by [37] (Reproduced with permission from [37]. Copyright, Springer, 2007). (**f**) Magnified image of the blue dashed frame in (e). The solid red line approximately parallel to the *z* axis passing through the tip peak shows the projection of the front tip edge at the cross section. The red circular area represents an arbitrary heating area on the heated tip surface few micrometers close to the tip apex. The scale bar is given in the lower right corner.

**Figure 4:**
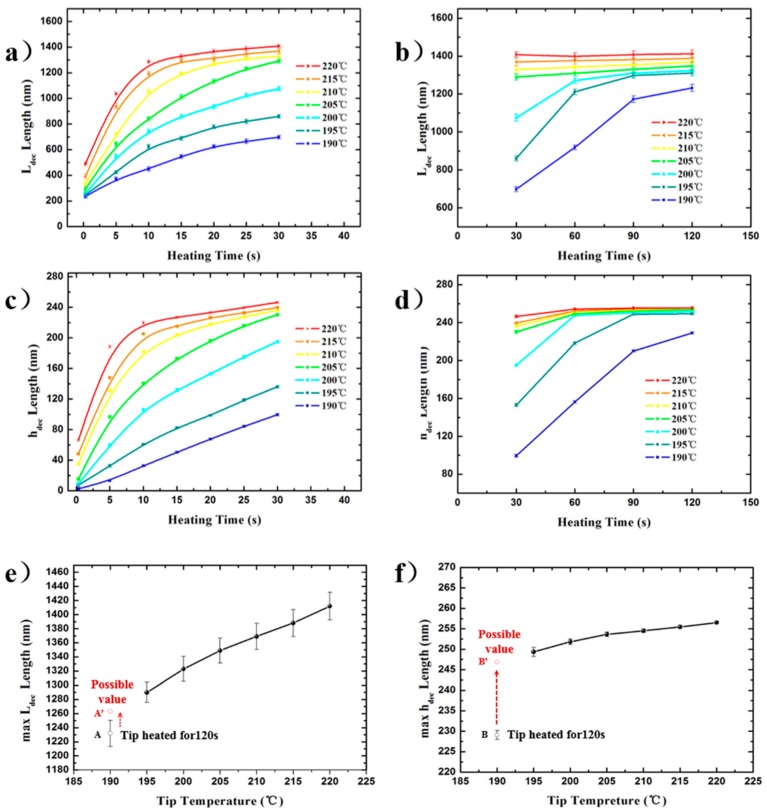
Function plot of the air gap feature sizes increasing with heating duration at the preset tip temperatures (*T*_t_) and the maximum feature sizes increasing with preset tip temperatures. (**a**)/(**b**) and (**c**)/(**d**) for *L*_dec_ and *h*_dec_ with 0.3–30 s/30–120 s as the varying of heating duration, respectively, at *T*_t_ of 190–220 °C. (**e**) and (**f**) for the max*L*_dec_ and max*h*_dec_ with a *T*_t_ of 190–220 °C.

The feature sizes and the formation process of the decomposition structure (air gap structures) induced by the heated tip were investigated in detail to analyze the NTFD in the nano/microscale air gap. The topography of the nano/microscale pyramid air gaps obtained in the heat-induced decomposition (see the experimental section for details) on the PPA film is shown in Figure 2a. The two feature sizes (*L*_dec_ and *h*_dec_) of the air gap in the PPA film, which formed under the preset tip temperature (205 °C) and varied preset heating time periods (0.3–30 s) are presented in Figure 2b. The formation process and topological characteristics of the air gap structures are shown in Figure 3. The evaluation of the feature sizes formed as a function of Tt and heating time provides insight into the horizontal and vertical evolution of Td in the form of an isotherm surface (Figure 4a–d).

Figure 2 shows the AFM topographic image of the pyramid decomposition structure fabricated on PPA film and the height cross-section plot of the marked location. The cross-section contours of the fabricated structures at the preset tip temperature of 205 °C or for the heating duration of 90 s were chosen as examples to show the intuitive variation of the two air gap feature sizes and the measurement method for *L*_dec_ as well as *h*_dec_. Obviously, with the extension of heating duration, the increasing speed of the value of feature sizes decreased gradually, and the size of the structure tended to be stable (Figure 2b) when the tip temperature was held constant at 90 °C. Therefore, the heating duration was extended to 120 s under each experimental temperature to verify whether there was a limit to the increase in the air gap dimensions with an increase in the heating time. Then, the decomposition structures fabricated under a longer heating duration were obtained (Figure 2c). When the tip temperature was set as 205 °C, the values of *L*_dec_ and *h*_dec_ with heating durations of 60 s, 90 s, and 120 s were 1.310 μm/249.2 nm, 1.320 μm/252.2 nm, and 1.349 μm/253.4 nm, respectively. As shown in Figure 2c, after a certain heating duration (reaching 90 or >60 s), the feature sizes of the obtained structures were nearly the same and their values did not increase at a prescriptive tip temperature. The maximum values of the air gap feature sizes were obtained at the condition of different preset tip temperatures with the heating duration of 90 s (see Figure 2d). Our other investigation showed that the formation and domains of the decomposition structure were related more to the tip temperature and the relative distance between the heated tip and the PPA film surface, less to the preset normal load on the cantilever, and negligible to the cantilever orientation (this refers to the angle between the direction of the cantilever leg and the *x*-axis).

Figure 3a–d show the AFM images of the decomposition-structures with the dimension extending from zero to the max value at the tip temperature of 205 °C. At the beginning (heating duration of 0.3 s) of the heat-induced decomposition of PPA, the heat transfer from the tip to the sample surface caused an expansion structure of 48–60 nm in height surrounding the area of the tip–sample initial contact (Figure 3a,g), which would increase the degree of the heated cantilever bending. Thus, the force feedback was switched off to avoid the relative position change of the heated tip and the sample surface caused by the material expansion. For the increasing heating duration, the surface contour of the formed air gap decomposition structure was not a symmetric rhombus cantered on the initial contact point (Figure 3b). However, the deviation from the symmetric rhombus always appeared on the side that was further away from the cantilever fixed end (area bordered by red dash lines in Figure 3b), which is a different phenomenon from the simulation and experimental results of the interface heat transfer in previous studies [31,33]. 

The deviation is attributed to the difference in the distance between the sample surface and a certain heating area on the heated tip surface (Figure 3e), specifically for three reasons. First, according to previous studies [9,35,36] on the tip–sample heat transfer, the heat flux *q*_gap_ from different areas of the heated tip surface to the sample surface is the most important factor to affect the distribution of the temperature field in the surrounding space of the initial contact point. This factor directly influences the occurrence region of thermal induced PPA decomposition. Specifically, the heated tip induced decomposition structure only formed below the tip parts that were close to the tip peak and from which the distances were less than 1.64 μm to the sample surface (Figure 3f). Second, the tilt angle of the cantilever to the substrate has been reported to be about 12 deg [30,34,37] and its value will increase with the bending back of the cantilever under a preset normal load when the material underneath the heated tip was decomposed (Figure 3e). The separation distance of the tip parts to the sample surface on either side (near or far from the cantilever fixed end) of the tip changed with the angle value, which could cause the surface temperature variation. Third, the tip shape may be another important factor for the deviation, and the analysis was conducted as follows. After the decomposition-structure dimension extended to a value approximately not changing with the heating time (Figure 3c,d). The distances between the initial contact point to the two edges along the y axis were *k*_1_ and *k*_2_, and their measured values were 1174 and 385 nm, respectively (Figure 3j). These values were approximately equal to the distance (1320/373 nm) between the z axis through the tip peak point to the point on the tip left/right edge located on the z axis through the left/right-edge point of the air gap decomposition structure (see Figure 3f). Therefore, under the condition of air gap heat transfer, the deviation phenomenon different to the previous results under the condition of the interface, and the substrate heat transfer conforms to the above three reasons.

Figure 3c shows some of the convex structures on the edge of the pyramidal air gap marked by the pink and green lines. Their maximum height were less than 60 nm and their maximum surface span was less than 600 nm (Figure 3h–i). The shape of their cross section was nearly the same as that of the expansion structure (Figure 3g). Thus, in combination with the experimental process (see heated-induced decomposition for details), the formation of these convex structures is due to the movement of the incomplete cooling tip on the sample surface, but not attributed to the heat transfer from the heated tip in the decomposition process.

Figure 4a–d show the plots of *L*_dec_/*h*_dec_ as a function of the heating time (*t*) for different preset tip temperatures (*T*_t_). The measured values of *L*_dec_/*h*_dec_ ranged from 235 ± 13.8/6.6 ± 2.0 nm to 1412 ± 19.6/256.5 ± 1.5 nm for 0.3 s at 190 °C and 120 s at 220 °C, respectively. The increasing tendencies of both feature sizes (*L*_dec_/*h*_dec_) at different preset temperatures were the same: increasing with the heating time, reached a plateau value (max*L*_dec_ and max*h*_dec_), in addition, at the tip temperature of 190 °C, the plateau value was still not reached. However, the rate of each *L*_dec_ to approach its maximum value was higher than that of each *h*_dec_ at any preset tip temperature. Specifically, for example, when *T*_t_ = 220 °C, max*L*_dec_ and max*h*_dec_ were 1412 ± 19.6 nm and 256.5 ± 1.5 nm, respectively. At *t* = 10 s, *L*_dec_ and *h*_dec_ were 1286 ± 16.9 and 219.7 ± 1.7 nm, which were ~91% and ~86% of their maximum values, respectively. Results showed that the heat transport in the horizontal direction may be greater than that in the vertical direction for certain time periods in the process of reaching their respective maxima. Specifically, according to the previous simulation results [38,39], when the tip–sample distance is in the nanoscale (<100 nm), the air medium in the gap cannot be regarded as a continuous medium, and the whole process is more like heat transfer in vacuum, there is a very remarkable phenomenon of thermal radiation. This sharp radiative exothermicity leads to a sharp increase in the material decomposition rate, which is one of the two reasons why in Figure 4a,c, at the beginning of the air gap decomposition, the curve shows a decreasing growth rate. Another reason is that when the dimension of the air gap structure is nanoscale at the beginning of the air gap expansion, the scale effect has a significant influence on the thermal conductivity of the discontinuous air medium [40]. This means that there is an anisotropy of the thermal conductivity in the x and z axis directions. Therefore the above statement is scientific. 

However, as shown in Figure 3d,j, the distance of the lateral morphology edge of the decomposition structure along the +*Oy* and −*Oy* direction from the tip peak was quite different (*k_2_* ≈ 3.05 *k_1_*). However, the formation of this result was different from the nature of the thermal conductivity anisotropy in the x and z axis directions as above-mentioned. The asymmetry is more likely due to the fact that all of the different regions on the tip parts close to the tip peak above the air gap can be regarded as heat sources, which can provide a thermal effect on the PPA material surface, as shown in Figure 3f. Therefore, the deviation in the morphology does not account for the difference in the thermal conductivity of the air media in the air gap in the two lateral dimensions. The existence of a plateau value of *L_dec_* and *h_dec_* in Figure 4b,d suggests that the feature size changing with the heating time is nearly constant. The phenomenon shows the process of the system heat exchange gradually reaching a quasi-steady state. As shown in Figure 4e,f, the max*L*_dec_ and max*h*_dec_ are represented as curves with temperature and they show an approximately linear changing tendency with respect to temperature. Points A and B are the experimental feature size values (Point A for *L_dec_*, Point B for *h_dec_*) of the air gap obtained at 190 °C and heating for 120 s. Since these feature sizes did not reach the plateau value with the increase in heating time, the actual maximum feature sizes should be higher. Moreover, according to the quasi-linear law obtained from other experimental points in Figure 4e,f, the possible maximum values corresponding to 190 °C may be in the positions of A’ and B’, as shown in Figure 4e,f.

For the changing tendency of the two feature sizes, in the increase interval, the higher the tip temperature, the steeper the temperature gradient in the air gap. The pyrolysis material response to the short-range temperature field that arises from the heated cantilever and extends into its surrounding nanoscale air gap during 3D patterning was in accordance with the previous research [3]. However, in the above process, the moving speed of the heated tip will affect the temperature field by influencing the tip-staying duration close above the material surface. It can be confirmed that the maximum range of the isothermal surface of 188 °C in the air gap temperature field is horizontal at ~700 nm (half of the max*L*_dec_) and vertical at ~250 nm under no movement of the heated tip in this study. The values of max*L*_dec_ as well as max*h*_dec_ at different preset temperatures were very close and showed a linear relationship, which indicates that the steady-state NTFD around the tip was consistent and was not affected by the tip temperature (*T*_t_ > *T*_d_) and the heat conduction from the tip to PPA material was characterized by one-dimensional heat transfer in the direction perpendicular to the air gap surface, respectively. Since the decomposition air gap structure was not hemispherical but pyramidal, the temperature field distribution in the air gap should be influenced by the tip shape close to the tip peak, which is consistent with the above analysis of the characteristics of the formation process of the decomposition structures. In addition, the expansion rate of the thermal field in the air gap around the heated tip in the horizontal direction along the material surface was higher than that in the vertical direction pointing to the material inner side, which indicated that the accuracy of the vertical analysis of the thermal field distribution was higher than that of the horizontal analysis.

### 3.2. Simplified Steady State 3D Model

From the generation to the expansion of the air gap structure, the heat conduction process is quite complex and cannot be analyzed with a single heat source. The whole heat conduction process includes:

(1) Contact heat transfer: contact heat transfer at the tip–sample interface causes surface decomposition of the PPA material and produces a nano-scale air gap.
(2) Thermal radiation: when the tip–sample distance is in the nanoscale at the beginning of the formation of the air gap, there is a very remarkable phenomenon of thermal radiation. However, the exact effective dimension of the thermal radiation range is hard to determine.
(3) Convection heat transfer: nanomaterial PPA decomposition is accompanied by solid–gas phase transition. When the air gap structure expands, there will be a convective heat transfer layer on the surface of the material, the thickness of which is related to the decomposition speed.
(4) The latent heat of phase transition: the solid–gas phase transition on the surface of the PPA material is always in existence. However, it has not been carried out under equal pressure in the gradually expanding micro-nano-scale air gap. Therefore, the latent heat of phase change is difficult to calculate by the time derivative of the volume change of the material.
(5) Air gap medium heat transfer: in all of the heat transfer processes described in 2), 3), and 4), there has always existed a heat conduction of the heated tip to the PPA surface through the sub-continuous air medium, whereas the thermal conductivity of the air medium due to the nanoscale effect is most likely anisotropic in the air gap space, early in the heating time. Moreover, the heat transfer through air is shown to be dependent on the sample thermal conductivity [41].

Therefore, the heat conduction during the formation and expansion of a micro-nanoscale air gap is too complex to undertake a comprehensive and accurate calculation about the NTFD. However, as above-mentioned, the existence of a plateau value of L_dec_ and h_dec_ in Figure 4b,d suggests that the process of the heat exchange gradually reaches a quasi-steady state. Based on the steady-state system, it is reasonable to ignore the radiation heat transfer (after the air gap extends to a certain size, the heat radiation effect can be ignored), ignore the convection heat transfer, ignore the latent heat of phase transition, and only focus on the analysis of the existing air gap medium heat transfer. Based on the experimental results, the maximum feature sizes of the decomposition structure were almost unchanged with the preset tip temperature, and the tip heat source was not equivalent to a hemispherical structure that is widely used in tip modeling, but to the very small part near the spherical tip peak with an asymmetrical pyramidal geometry shape.

**Figure 5:**
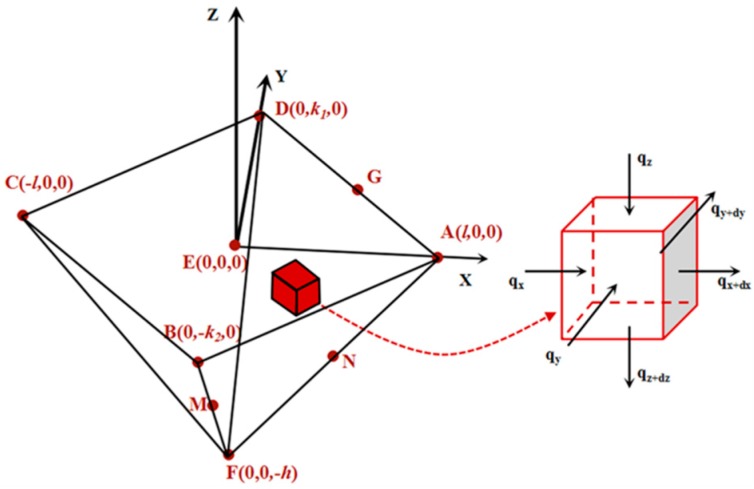
Simplified 3D model of the temperature field in the pyramid nano/microscale air gap is shown on the left side. The coordinate system is located at the initial contact point. Points A, B, C, and D are the vertex of the surface contour; point E is the position of tip peak approximate coincidence with the origin; point F is the vertices of inverted pyramid air gap structure; points G, M, and N are certain points on their own pyramid edge. The red cube is a heat conduction element at any position inside the temperature field. The thermal equilibrium analysis of the heat conduction element is shown on the right side.

By determining the relative position between the 188 °C isothermal surface and tip source in the air gap temperature field (category I definite solution condition), we can calculate the steady-state thermal field distribution around the tip at a certain tip temperature. The obtained air gap decomposition structures were represented by the two feature sizes to make the analysis of NTFD more convenient. All the isothermal points on the known isothermal surface of the air gap decomposition structures around the heated tip were taken into account in a simplified 3D steady-state pyramid heat conduction model (see Figure 5).

The model is based on the following hypothesis:

(1) The system is in a steady-state;
(2) The very small part near the spherical tip peak with a pyramidal geometry shape and a preset temperature *T*_1_ can be regard as the heat source of air gap heat transfer;
(3) The latent heat of cooling on the convective surface can be neglected [33]; and
(4) Phase changes can be neglected.

Based on the Laplacian energy conservation equation [42], we derived the equation, which is shown as follows:

(1) $$\frac{\partial^{2}T}{\partial x^{2}}+\frac{\partial^{2}T}{\partial y^{2}}+\frac{\partial^{2}T}{\partial z^{2}}=0$$

where *T* is the temperature at point (*x, y, z*). In the steady-state thermal field, the heat flux is continuous at the interface, which is the surface of the pyramid decomposition structure.

(2) $${\left(\eta \frac{\partial t}{\partial n}\right)}_{I}={\left(\eta \frac{\partial t}{\partial n}\right)}_{II}$$

where *η* is the thermal conductivity of heat transfer media on either side of the interface I for air media and II for the PPA material media, and *n* is the unit vector perpendicular to the interface direction. 

According to Equation (1), the general expression of *T* can be expressed as follows:

(3) $$T=ax^{2}+by^{2}+cz^{2}+dxy+eyz+fzx+mx+ny+pz+t$$

where *a, b, c, d, e, f, m, n, p,* and *t* are the undetermined coefficients. Based on a quasi-steady state analysis, when Equation (1) is established, it can be inferred that:

(4) a+b+c=0

The determination of the above undetermined coefficients needs to be calculated and determined with boundary conditions. In the following, the point coordinates with determinable temperature are expressed as *(x, y, z; T_xyz_)*. The function value of T at the point *(0, 0, 0; T_0_)* is substituted into Equation (3), and *t* = *T*_0_ is obtained. As the probe material and its thermal conductivity are known [17], according to the preset tip temperature *T*_1_, *T*_0_ can be expressed approximately as *T*_1_ + 0.73 °C ± 0.26 °C, namely, *T_0_* ≈ *T_1_* + 0.73 °C ± 0.26 °C. According to assumption 2, there is an equivalent heat source related to the shape of the tip near below point E in the steady-state heat field. The preset tip surface temperature is *T*_1_. The point coordinates on its sphere satisfy the following equation:

(5) $$x^{2}+y^{2}+{\left(z+\lambda -r\right)}^{2}=r^{2}$$

where λ is the distance from the peak of the tip to the origin E, and *r* is the radius of the spherical tip peak. According to Equation (2), and the temperature continuity at the interface, any point P *(x, y, z)* satisfying Equation (5) is substituted into Equation (3), and *T* = *T*_1_ is found. For special selection points *(σ, 0, 0; T_1_), (0, σ, 0; T_1_), (0, 0, −λ, T_1_)*, the following three equations can be obtained:

(6) $$T_{1}-T_{0}=a\sigma^{2}+m\sigma =\Delta T_{1}$$

(7) $$T_{1}-T_{0}=b\sigma^{2}+n\sigma =\Delta T_{1}$$

(8) $$T_{1}-T_{0}=c\lambda^{2}-p\lambda =\Delta T_{1}$$

where *σ* is the intercepts of the spherical tip on the *x* and *y* axis. The position of the point on the outer surface of the air gap structure in the coordinate system satisfies:

(9) $$\frac{1}{l}x+\frac{1}{k_{1}}y-\frac{1}{h}z=1,\;\left(x>0,\;y>0\right)$$

(10) $$-\frac{1}{l}x+\frac{1}{k_{1}}y-\frac{1}{h}z=1,\;\left(x<0,\;y>0\right)$$

(11) $$-\frac{1}{l}x-\frac{1}{k_{2}}y-\frac{1}{h}z=1,\;\left(x<0,\;y<0\right)$$

(12) $$\;\;\frac{1}{l}x-\frac{1}{k_{2}}y-\frac{1}{h}z=1,\;\left(x>0,\;y<0\right)$$

Since the equation needs to be expressed in four intervals according to the value range of the variables, *x* > 0 and *y* > 0 were selected for subsequent derivation. Then, according to Equation (2) and the continuous temperature at the interface, *T* = *T*_2_ can be determined, if any point *P (x, y, z)* satisfying Equation (9) is substituted into Equation (3). For special selection points (*(l, 0, 0; T_2_)*, *(0, k_1_, 0; T_2_)*, *(0, 0, −h, T_2_)*, the following three equations can be obtained:

(13) $$T_{2}-T_{0}=al^{2}+ml=\Delta T_{2}$$

(14) $$T_{2}-T_{0}=bk_{1}{}^{2}+nk_{1}=\Delta T_{2}$$

(15) $$T_{2}-T_{0}=ch^{2}-ph=\Delta T_{2}$$

By combining Equations (6), (7), (8), (13), (14), and (15), Equations (16)–(18) can be obtained:

(16) $$a=\frac{\sigma \Delta T_{2}-l\Delta T_{1}}{l\sigma \left(l-\sigma \right)},$$

(17) $$b=\frac{\sigma \Delta T_{2}-k_{1}\Delta T_{1}}{k_{1}\sigma \left(k_{1}-\sigma \right)},$$

(18) $$c=\frac{\lambda \Delta T_{2}+h\Delta T_{1}}{h\lambda \left(h+\lambda \right)},$$

Substitute Equations (16)–(18) with respect to a, b, and c into Equation (4) to obtain:

(17) $${\Delta T}_{2}=F\left(l,k_{1},\sigma ,h,\lambda \right){\Delta T}_{1}$$

The partial derivative is obtained from Function *F* with respect to *l*. When *l > 10 σ*, there exists:

(20) $$\underset{l>>\sigma }{\lim}\frac{\partial F}{\partial l}=\frac{1}{\sigma }$$

Therefore, when *l* > 10, there exists:

(21) $$\frac{{\Delta T}_{2}}{\Delta T_{1}}\approx \frac{l}{\sigma }$$

Equation (21) indicates that the *x* axis scale of the isothermal plane is proportional to the change in temperature of the tip when it is expanded to a certain range (*l >>* σ). This conclusion is also consistent with the experimental results, as shown in Figure 4e. Similarly, the same is true for the y and z axes. Substitute Equation (21) into Equation (16), and at *l >*10 *σ*, there exists:

(22) $$\underset{l>>\sigma }{lim}a\approx 0$$

Therefore, in the *x* direction, when the air gap feature size *l* is much larger than the circular radius of the cross section of the tip sphere (conformation to the scale obtained in the experiment), the weight of the quadratic term is very small. By the same logic, it is can be known that the direction of *y* and *z* is also true. Therefore, under the restriction of the boundary condition (Equation (2) obtained in this paper), Equation (3) can be expressed as:

(23) T≈mx+ny+pz+t

In the four quadrants divided by the *x* and *y* axes, boundary condition Equations (9)–(12) were substituted into Equation (23), and then generalized and unified to obtain:

(24) $$\frac{T-T_{1}}{T_{2}-T_{1}}=\frac{1}{l}\left|x\right|+\frac{1}{k_{i}}\left|y\right|-\frac{1}{h}z\;,\left(i=1,2\right),$$

where *T*_1_ and *T*_2_ are the preset tip temperature and the air gap surface temperature, respectively. Since the temperature on the boundary is continuous in the steady thermal field, *T*_2_ is always equal to the PPA *T*_d_ (188 °C) and *T*_1_ > *T*_2_; *h*, *l*, and *k* are the max*h*_dec_, half max*L*_dec_, and *k*_1_ (*y* > 0) or *k*_2_ (*y* < 0) of the decomposition structure at the preset temperature *T*_1_, respectively. The definition of *k_i_* (*i* = 1, 2) is given in Figure 3j. The max*h*_dec_, max*L*_dec_, and *k_i_* (*i* = 1, 2) should follow the corresponding relation determined by the asymmetry of the decomposition air gap structures based on the experimental facts and the mounting angle of the cantilever, which means that when two of the three values are identified, the left one can be calculated. In the condition that the tip temperature is between 190 to 220 °C, all *h*, *k_i_* (*i* = 1, 2), and *l* in the steady-state temperature field have the following correspondence:

(18) $$k_{1}\approx \frac{lh}{l–h}\;,$$

(19) $$k_{2}\approx 3.05k_{1}\;,$$

Thus, the distribution of the temperature field can only be calculated directly based on the measurement of the max*L*_dec_ and max*h*_dec_. Since the two feature sizes of the air gap structures had an approximate linear relationship with the tip temperature, at a certain tip temperature, when the heat conduction in the air gap reaches the steady state, the feature sizes can be determined, and the temperature field distribution at this temperature can be calculated quantitatively by combining Equations (24)–(26). However, the first two feature sizes required to calibrate the isothermal surface of the decomposition threshold temperature can only be measured directly through the experimental tests.

**Figure 6:**
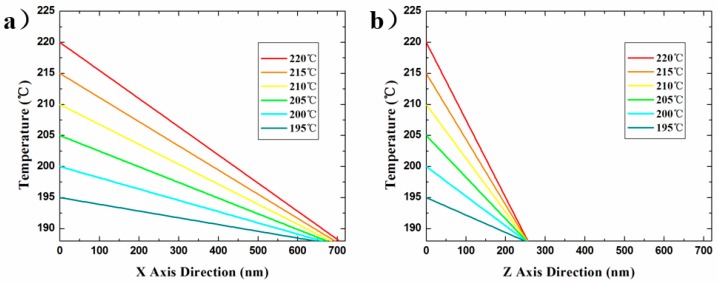
Calculated steady-state temperature profiles of (**a**) x axial and (**b**) z axial direction from the heated tip to the sample surface in the pyramid air gap. The preset tip temperature expressed in different colors is from 195 to 220 °C. The temperature is expressed as a function of the distance away from the tip, and the slope of the curve represents the temperature gradient at any point on it.

Figure 6 shows a decreasing tendency of the temperature from the tip to the surrounding air gap under any preset tip temperature, which was calculated by our model. The results indicate that the higher the preset temperature, the faster the temperature drops. However, the temperature of the point in the air gap with a certain distance away from the tip all tends to the decomposition-temperature threshold of the material at the same location such as ~670 nm along the x axis or ~250 nm along the z axis, regardless of the preset tip temperature range from 195–220 °C. Though the value of the above distance is not possible to calculate effectively without experiments, it might be qualitatively predicted by analyzing the following four influencing factors: (i) the geometry of the tip a few micrometers close to the tip peak point; (ii) the thermal conductivity of the heated-tip material; (iii) the thermal conductivity of the medium composition in the air gap; and (iv) the thermal conductivity of the medium composition in the air gap. A promising research direction involves exploring the above factors in order to determine the temperature distribution of the probe tip on the material surface by direct calculation.

**Figure 7:**
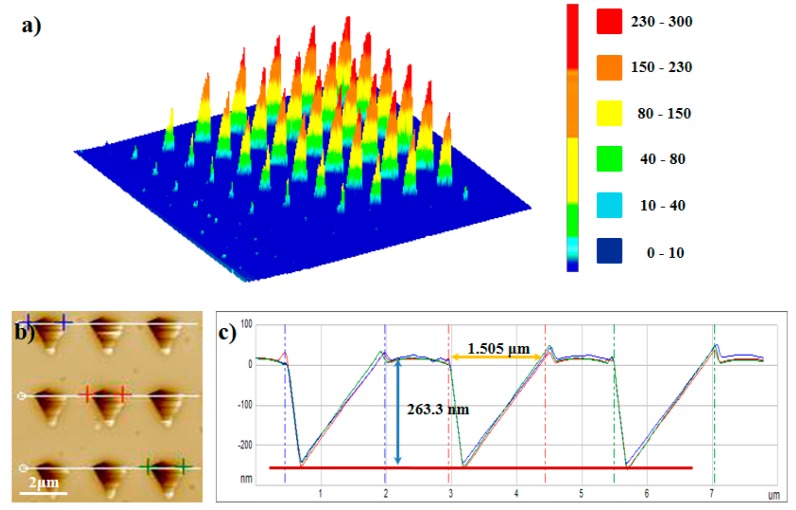
Patterning pyramid structure on the PPA film. (**a**) Three-dimensional image of pyramid structures (in Figure 2a). (**b**) AFM topographic image of patterning structures. The heating time was 30 s, and the tip temperature was 245 °C. (**c**) The height cross-section plot of the position marked by the blue/red/green line in (b). The size and shape of every pyramid decomposition structure were all the same. The feature sizes of the structure, max*L*_dec_ and max*h*_dec_ were 1505 ± 18.4 nm and 263.3 ± 1.9 nm, respectively.

The simplified model could not completely simulate the heat conduction process, but could predict the thermal gradient relatively. The above calculation results in Figure 6 revealed the temperature field distribution at <700 nm (horizontal) or <250 nm (vertical) away from the heated tip. Since the tip shape, material properties, and medium properties are not involved in Equation (24), the steady-state temperature field distribution at the preset tip temperature can be calculated only by using the air gap feature sizes. The temperature distribution of the nano/microscale air gap in the application or investigation of pyrolytic materials can be quantitatively calculated by using the simplified model based on our experimental method, without detailed measurement of the four influencing factors above-mentioned. This extremely simple calculation cannot match the whole complex heat transfer problem. However, this model has a strict premise of the use, that is, in the thermal steady state. Moreover, this simplified 3D computational model, in the temperature range of further concern, is indeed able to predict the extent to which the nano gap size continues to expand, as shown in Figure 7. We used experimental methods to verify the validity of our computational model: using the above simplified steady state 3D model, assuming that the tip temperature was 245 °C, the calculated values of two feature sizes of the isothermal surface of 188 °C in the temperature field were 1513 ± 25.7 nm and 264.8 ± 2.0 nm, and the actual experimental measurement results under the experimental conditions in this study are shown in Figure 7b, which matched well with the above calculated results.

The results in our paper can be used in future studies to further improve the heat transfer model of a thermal AFM cantilever hovering close above the pyrolytic polymer surface. Our experimental method could provide the verification for the simulation results of NTFD in the nano/microscale air gap around the heated tip and our simplified model is a useful simplification for the investigation of the complex heat transfer problems in the nano/microscale air gap. More importantly, although the thermal probe tip geometry and electro-thermal conversion ability used by each scholar or research group may be different, even the tip geometry of one thermal probe may change because of the tip wear, so the nano-material PPA for the nano-air-gap decomposition experiments described in this paper can be used to obtain the corresponding air gap structures that fits the selected probe. After obtaining these kinds of air gap structures, scholars can design the machining structure size by measuring the feature sizes and calculations by using our model. It is worth particularly mentioning that, by using the simplified model, a type of nano/microscale structures with programmed sizes could be fabricated by controlling the heated tip, inducing PPA surface decomposition. The surface profile accuracy of the structures was proven to be less than 20 nm. This is a new type of nanostructure fabrication method using SThM. As shown in Figure 7a, when the cantilever is hovering close above the PPA surface, the dimensions of the formed decomposition structures can reach a level of 10^0^ μm, which is a new way of fabricating microscale structures directly using thermal probe. The consistency of the feature sizes of the obtained structures shows the stability of this fabrication method (Figure 7b), which may be a potential application of an AFM heated tip by controlling its thermal field distribution. Therefore, based on the above considerations, we think that the establishment of this simple 3D air gap heat transfer model for NTFD of great value.

## 4. Conclusions

Using the SThM technique and AFM topography, the thermal transfer and the temperature field distribution in the nano/microscale air gap were investigated through the determination of the formation process and the feature sizes of the heated induced air gap decomposition structures on the PPA material. The results are as follows:

(i) Different nano/microscale pyramid air gaps with an approximately rhombic surface contour were formed on the surface of the PPA film by the thermal effect of the heated tip with its location holding at the initial contact point and temperature ranging from 190 to 220 °C for the various heating durations (0–120 s). The air gap horizontal and vertical dimensions ranged from 235 ± 13.8 to 1412 ± 19.6 nm and 6.6 ± 2.0 to 256.5 ± 1.5 nm, respectively.
(ii) The heat transfer in the horizontal and vertical directions was estimated from the variable air gap dimensions. In the air gap between the heated tip and sample surface, the horizontal was greater when compared with the vertical heat transport, and the horizontal thermal transfer was not always symmetric and the deviation appeared on the further side from the cantilever fixed end, which was determined by the air gap heat transfer mode, tip geometry shape, and the cantilever mounting angle. The position of the 188 °C isothermal surface of the steady-state temperature field in air gaps was quantitatively determined by our experimental method. The maximum space range of the air gap temperature field in our investigation was horizontal at ~700 nm and vertical at ~250 nm.
(iii) A simplified model of the 3D steady-state thermal field for the nano/microscale air gap was established and the boundary condition was obtained by measuring the feature sizes of the air gap structures. The temperature distribution could be calculated by the model, and the results showed that the higher the preset tip temperature, the steeper the temperature gradient close to the tip peak. By using our simplified model, the temperature distribution could be quantitatively calculated with only the measurements of the air gap feature sizes without measuring the tip geometry shape, the cantilever mounting angle, and the system (heatable tip, medium, and sample material) heat conduction characteristics, which is a useful simplification for the investigation of complex heat transfer problems in the nano/microscale air gap.
(iv) Based on the calculation of our model, we fabricated programmable nano/microscale (20–1500 nm) pyramid structures on the PPA film. The structure dimensions were designed by controlling the temperature field around the heated tip. The processed structures had great consistency and repeatability, thus may be a potential application of nanofabrication based on SThM.
